# Deep Reads: Favorites from a Few Different Shelves

**DOI:** 10.1371/journal.pgen.1006476

**Published:** 2016-12-15

**Authors:** Bruce A. Hamilton

**Affiliations:** Department of Cellular and Molecular Medicine, Division of Medical Genetics, and Institute for Genomic Medicine, University of California, San Diego School of Medicine, La Jolla, California, United States of America

One of my favorite traditions is giving books to students who graduate from my lab or complete the training program I run. This has come to encompass a wide range of titles to suit a variety of science interests. For this column, I selected some favorite genetics and genetics-related books from different shelves of my own collection that have not been covered by the excellent recommendations earlier in this series [1–3]. While some are out of print, used and library copies preserve all the same pleasures to read again (Fig 1).

**Fig 1:**
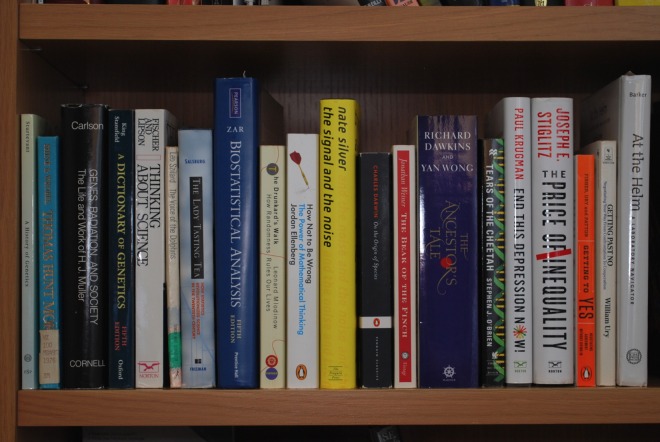
Some books the author has enjoyed. Image courtesy of Bruce Hamilton.

## A Look Back

Anyone interested in the origins of the field should have (and read) A. H. Sturtevant’s ***A History of Genetics*** (1965). This succinct account begins with a description of the thinking on heredity that existed before the 1900 rediscovery of Mendel’s paper and then follows the aftermath, giving us an inside view into how early investigators framed the questions, the experiments that gave key answers, and the eventual resolution of some apparent exceptions. As Ed Lewis notes in his introduction to the 2001 edition, it can be instructive to consider how controversial were some of the foundational ideas that we now accept as obvious and how well-accepted, in at least some quarters, were ideas that we now consider foolish. An excellent curation of original papers mentioned in the book, along with other important papers from the early decades of genetics and an electronic version of the book itself, is available from Electronic Scholarly Publishing (at the prescient URL, www.esp.org).

T. H. Morgan, Sturtevant’s mentor, was a founding father of genetics and the field’s first Nobel laureate. Morgan’s story is told in two biographies: ***Thomas Hunt Morgan*: *Pioneer of Genetics*** by Ian Shine and Sylvia Wrobel and ***Thomas Hunt Morgan*: *The Man and His Science*** by Garland E. Allen. A Kentucky native, Morgan occupied an interesting confluence of American history and American science. He was a great-grandson of Francis Scott Key, a nephew of Confederate General John Hunt Morgan, and distant cousin of the financier John Pierpont Morgan. He earned the only bachelor of science degree from the State College of Kentucky in his graduating class, which comprised just two students. He went to graduate school “because he didn’t want to go into business and didn’t know what else to do” (an explanation that no longer works in an admissions essay). Studying at Johns Hopkins, Morgan came into an intellectual pedigree that Sturtevant traces back to Immanuel Kant, through W. K. Brooks and Louis Agassiz. It was also at Hopkins that Morgan met Edmund B. Wilson, who would be instrumental in bringing Morgan first to a teaching position at Bryn Mawr and then to Columbia University, where he would begin the *Drosophila* work that is recounted in both biographies and in Sturtevant’s *History*. As Morgan neared the mandatory retirement age at Columbia, he chose to relocate instead. He brought a vision to put genetics on a physical basis–as well as Sturtevant, Bridges, and other members of the fly room–to a new Division of Biology at a young institute in California. The success of Morgan’s Division of Biology at Caltech may tell us something of what cast-offs can do in a favorable environment.

In 25 years at Columbia, Morgan taught introductory biology only once. From this class, he recruited two remarkable students to his laboratory: Sturtevant (who would succeed Morgan as Chair of the Division of Biology at Caltech) and Calvin Bridges (whose hand-drawn polytene chromosome maps were a staple in *Drosophila* labs before being superseded by George Lefevre’s photographs). They both joined a Biology Club started by Hermann Muller, who would then follow them into Morgan’s lab (and would follow Morgan to a Nobel prize in 1949 for his discovery of X-ray mutagenesis). Bridges may have been the most vivid personality among the three, but Muller’s biography is the one I come back to whenever I feel things are not working out as they should.

Elof Axel Carlson’s magnificent volume, ***Genes*, *Radiation*, *and Society*: *The Life and Work of H*. *J*. *Muller***, is a masterclass in scientific biography. Alongside the seminal insights and discoveries of Muller’s scientific career, Carlson shows how Muller interacted with and reacted to the world around him, including most of the upheavals of the 20th century. Muller became a key member of the fly room at Columbia, yet felt unappreciated within it. He moved to an independent position in Texas and married a young math professor there, who was then terminated for being a married woman. After some tumultuous years that included a temporary return to New York during World War I, a new position in Austin, a suicide attempt, and a 1932–1933 sojourn in Germany, Muller took up Nikolai Vavilov’s invitation to move to the USSR. After a divorce, Muller’s ex-wife returned to Texas and married his former student. After Vavilov was purged by Stalin, Muller negotiated an exit from the Soviet Union by volunteering as a medic for the Red Brigade in the Spanish Civil War. Muller then took a temporary position in the United Kingdom, arriving in Edinburgh on the eve of World War II with an expertise in radiation genetics and an interesting passport history. It doesn’t slow down from there.

The history of genetics is also reflected in terms of art that we use. As Ed Lewis notes in the afterword to *A History of Genetics*, even Sturtevant and Beadle differed in how each used the word “gene,” to mean either a variant or a locus [4]. ***A Dictionary of Genetics*** by Robert C. King and William D. Stansfield is an indispensable guide for avoiding the drift in meaning that often comes from frequent and sometimes incautious usage. I consult my copy often for writing, to increase the likelihood that I am using terms in a way others will easily recognize. I will simply mention the word epigenetics and hide.

Andrew Chisholm covered the golden era of phage genetics in this space two years ago [3], and I highly recommend that reading list and one addition. In ***Thinking About Science***, Ernst Peter Fischer and Carol Lipson provide a detailed and warm biography of Max Delbrück, one of the spiritual leaders of the phage church, whose impact on modern biology would be hard to overstate. The book covers his childhood in a prominent German family, his contributions to theoretical physics in an age of giants, and his turn to phage as the atom of biology. The clarity with which Max conceptualized a problem, the rigor with which he examined the evidence, and his willingness to revise his views in response to new evidence provide an elegant example for any young (or not so young) scientist. Max always advised his students, “Don’t do fashionable science.”

As technology continually reshapes how we live and work, fiction can be an excellent vehicle to identify the several ways we might feel about it. Leo Szilard’s 1961 short story collection, ***Voice of the Dolphins***, is still a good read for this reason. The thin and prescient volume includes “The Mark Gable Foundation” (1948), which I first became aware of through Peter Lawrence’s column [5]. In the story, a time-traveling scientist advises a wealthy benefactor on how to slow the pace of science: by endowing grants. Appointing the most productive scientists to prestigious review committees would remove them from their laboratories, he argues, while forcing other scientists to focus on fashionable topics in order to get the grants. By chasing fashions, nothing substantially new will get done. One wonders how he might have felt about citation metrics.

## By the Numbers

Genetics is inherently a quantitative science and is a sibling field to statistics in many respects, including shared lineage through Francis Galton (Darwin’s half-cousin), R. A. Fisher, and others.

***The Lady Tasting Tea*: *How Statistics Revolutionized Science in the 20th Century*** by David Salsburg is my personal testament to browsing in bookstores. If a lay history of statistics sounds dry, you may be surprised by how lively the story becomes in Salsburg’s telling. I often felt lost in the forest of null hypothesis tests and the underbrush of names attached to them before stumbling across this book. I was shopping for my dad. Glancing at the title, I thought, “probably dull.” It isn’t. Salsburg makes a compelling story of the concrete problem that each new advance was designed to solve. From Galton’s biometrics and Mr. Gossett (Student)’s *t* test—for sampling yeast concentration to brew Guinness more consistently—to Efron’s bootstraps and other computational advances, these are fascinating vignettes of both the problems for which many popular statistical methods were devised and the people who did the devising. This illuminated statistical methods for me in a way that more didactic books never did. When I realized I had spent 20 minutes absorbed in Salsburg’s book while making myself comfortable on the floor, I got up and bought two copies. Having read this history has made it much easier for me to fill in gaps in my own statistical training, often with Jerrold Zar’s excellent textbook ***Biostatistical Analysis***.

***The Drunkard’s Walk*** by Leonard Mlodinow is another engrossing read, focused on probability, uncertainty, and recognizing randomness. An example that will be familiar to some readers is the Monty Hall Problem, which Mlodinow paraphrases:

Suppose the contestants on a game show are given the choice of three doors: Behind one door is a car; behind the others, goats. After the contestant picks a door, the host, who knows what’s behind all the doors, opens one of the unchosen doors, which reveals a goat. He then says to the contestant, “Do you want to switch to the other unopened door?” Is it to the contestant’s advantage to make the switch?

If you aren’t familiar with the problem and do not see how its solution requires defining which processes in the set-up are random and which are not, then you owe yourself this book. Mlodinow gives one of the most engaging introductions to Bayes’ theorem, including his personal story of a positive HIV test and his relief upon learning that the false positive rate was “only” 1/1000. The final chapter discusses how an apparent run of successes (or failures) can be conjured from a string of random events by nonrandomly selecting a string that fits the desired narrative among many that do not—an important lesson for anyone investing in securities, hiring an executive, or betting on sports. A student to whom I gave this book commented, “From the title I thought you gave it to me as a joke, but I really enjoyed it.”

***How Not to Be Wrong*** by Jordan Ellenberg is another great read in logic and quantitative thinking (recommended to me by Casey Greene, through Kevin Mitchell’s Twitter thread on books for students). Ellenberg articulates several terrific examples of framing the right question, such as Abraham Wald’s advice to armor planes in the places where the bullet holes weren’t, and finding the unexplored opportunity, such as investment cartels exploiting fluctuations in expected value of some lottery games to ensure a consistent profit from buying lottery tickets. The first 100 pages in particular, including stories of the Baltimore Stockbroker and Bible code scams, should be required reading. I bristled a bit at his discussion of genomics (weak papers have been published, but that some in a field initially set a low bar for their work is itself a low bar for critiquing a field), but this is a book I would read again (and probably will).

Nate Silver’s ***The Signal and the Noise*** details the FiveThirtyEight founder’s Bayesian approach to forecasting and other quantitative exercises. He begins with an analysis of some prominent failures, such as the United States housing bubble of the 2000s and the regular failure of political pundits’ predictions. Silver discusses his development of PECOTA, a forecasting system for baseball players based on the rich performance data available, and how the entry of new kinds of data requires redevelopment of analytical tools in a given field—a lesson not lost on those of us who predate whole-genome acronyms. Silver’s description of the online poker bubble and the conditional probability of an adequate margin in a rapidly adapting market is also memorable. The final chapter is a reminder that missing information can humble even the most careful models, whether forecasting terrorism or evaluating baseball fielding performance.

## A Few More Selections

Biological evolution and market economics share a formal mechanism in selection on multiple and often hidden variables. (How anyone can embrace one without acknowledging the other is one of the most remarkable intellectual contortions on regular display.) A few books in each field have my recent or renewed attention, beginning with a trio on evolution. Darwin’s ***On the Origin of Species*** is an excellent if obvious place to start. Darwin draws explicitly from Malthus’ *Principle of Population* in framing the struggle for existence. His discussion of domestication is also particularly worth returning to in light of the many recent papers examining the genomic effects of selection on many domesticated species. Jonathan Weiner’s classic ***The Beak of the Finch*** is an excellent complement, covering the decades-long work of Peter and Rosemary Grant to study the impacts of changing conditions on trait distributions, allele frequencies, and the likelihood of species hybrids in Darwin’s Galapagos finches. In ***The Ancestor’s Tale***, Richard Dawkins (with Yan Wong in the revised edition) takes a narrative device from *The Canterbury Tales* for a trek across evolution. We are joined by other species as we move back in time across progressively more ancient branches in the tree of life, each branch point holding our most recent common ancestor with the species we meet. I filled in a few of the gaps in my understanding of zoology and evolutionary relationships while being entertained by this volume, which also captured the interest of some of my nonbiologist friends and relatives. Dawkins’ reading of Darwin on the audiobook version of the *Origin* brings us full circle.

Another favorite chronicle of the battles among genetic variants is Stephen O’Brien’s ***Tears of the Cheetah***, subtitled *The Genetic Secrets of Our Animal Ancestors*. Each chapter is a different story told in a captivating style. My personal favorite is his telling of the hunt for genetic resistance to a fatal retrovirus in wild mice at Lake Casitas that led to identification of the *Fv4* (*Akvr*) host restriction locus, an endogenized viral envelope gene whose expression competitively blocks exogenous virus from its receptor.

In economics (my college minor somety years ago), Paul Krugman’s clarion ***End This Depression Now*** can be read as an account of contextual selection, here with respect to economic policy. He shows how optimal policy choices depend in sometimes-unexpected ways on prevailing economic conditions, as much as optimal finch genotypes depend on environmental conditions. One clear and recent example is how interest rates near the zero lower bound change incentives on borrowing and spending. Borrowing at negligible (or sometimes negative) interest rates for expansionary spending in a depressed economy is not at all the same as borrowing under more ordinary conditions to pay recurring bills. Successful economies, like successful biological lineages, adapt.

***The Price of Inequality*** by Joseph E. Stiglitz is somewhat wonkier and meatier, but a highly rewarding read relevant to current debates in economic policy. The main point, as suggested by the title, is that extreme resource allocations are also inefficient. Highly unequal growth generally offers slower overall growth and lower stability. While it is tempting to draw parallels between resource allocation among economic actors and species interactions in an ecosystem, Stiglitz reinforces a key difference: unlike ecosystems, economies reflect political choices that set both the terms of competition and the distribution of rewards for success. These choices matter and are subject to negotiation.

***Getting to Yes*** by Roger Fisher and William L. Ury (with Bruce Patton for the revised edition) is a classic on negotiation principles and strategy. Academic scientists negotiate all the time—whether with administrators, peers, trainees, or reviewers—but often without thinking about the interaction as a negotiation. The advice for principle-centered negotiation is mostly straightforward: know your best alternative to an agreement, develop those alternatives before you need to negotiate, and think of ways to meet both your interests and the other party’s beyond simple positions. Though familiar, having the points clearly articulated in this book has several times helped me to plan ahead and to make the best of situations where I hadn’t. When I was looking for advice on negotiating for my first faculty job, Leonid Kruglyak, then a fellow postdoc, recommended I read *Getting to Yes* first. Once I had read it, I sat down with our advisor, who began reciting chapter and verse. I said, “I’ve read that book.” He said, “Well, that’s all I know.” This brief and pleasant volume is probably not all Eric Lander knows about negotiation. But that is a pretty good endorsement. And its sequel, ***Getting Past No*** by Ury, wouldn’t hurt either.

Veering just a bit to management, ***At the Helm*** by Kathy Barker is a useful guide to running a lab for new (and not so new) Principal Investigators, based on her interviews with successful PIs. Like *Getting to Yes*, many of the suggestions are things you already know but may not think about explicitly, nor have clearly framed in context just when you most need to know them. Having them laid out plainly as they are here should help to keep focus as you build your version of “the lab where everyone wants to be.”

These are genetics (and genetics-adjacent) reads I have enjoyed over the last few years. For pure bibliophilia, I cherish a copy of ***Retroviruses*** that one of my students had Harold Varmus inscribe; a copy of François Jacob and Elie Wollman’s ***Sexuality and the Genetics of Bacteria***, stamped “S. E. Luria” inside the cover, that I bought for a dollar in the basement of the MIT library; and Mary Esther Gaulden’s former copy of ***Genes*, *Radiation and Society*** that I bought online. These are some of my favorites. What are yours?
